# Sorbitol accumulation decreases oocyte quality in aged mice by altering the intracellular redox balance

**DOI:** 10.18632/aging.203747

**Published:** 2021-12-12

**Authors:** Yuexin Zhang, Zhengjie Yan, Hanwen Liu, Lingjun Li, Chun Yuan, Lianju Qin, Lingbo Cai, Jiayin Liu, Yanqiu Hu, Yugui Cui

**Affiliations:** 1 State Key Laboratory of Reproductive Medicine, Clinical Center of Reproductive Medicine, First Affiliated Hospital, Nanjing Medical University, Nanjing 210029, Jiangsu Province, China

**Keywords:** sorbitol, *in vitro* maturation of oocytes, aged mouse oocytes, sorbinil, reactive oxygen species

## Abstract

Sorbitol is a product of glucose metabolism through the polyol pathway. Many studies have demonstrated that excessive sorbitol can disrupt the intracellular redox balance. However, we still know very little about the impact of excessive intracellular sorbitol on oocyte quality, oocyte maturation, and embryo developmental potential. This study explored whether intracellular sorbitol accumulates in the oocytes of aged mice during *in vitro* maturation (IVM) and what roles sorbitol plays in oocyte development and maturation. Our results showed that sorbitol levels were significantly higher in *in vitro*-matured oocytes from aged mice than in oocytes from young mice (14.08 ± 3.78 vs. 0.23 ± 0.04 ng/oocyte). The expression of aldose reductase (AR) mRNA was significantly higher in the *in vitro*-cultured oocytes from 9-month-old mice than prior to culture. To decrease the excessive intracellular sorbitol in oocytes from aged mice, sorbinil, a specific inhibitor of aldose reductase, was supplemented in IVM medium, and the sorbitol level was significantly decreased (14.08 ± 3.78 vs. 0.48 ± 0.19 ng/oocyte). Our results indicated that the percentage of oocytes with first polar body extrusion (PBE) was significantly higher in the sorbinil group than in the aged group (82.4% ± 7.2% vs. 66.1% ± 6.9%), and the content of sorbitol was drastically increased in the aged group. The ROS fluorescence intensity in the sorbinil group was drastically lower than that in the aged group, while the GSH fluorescence intensity was significantly higher. Interestingly, SOD1 was upregulated in the sorbinil group. The present study suggests that excessive sorbitol accumulation is induced during IVM in aged mouse oocytes, which negatively influences oocyte quality by altering the intracellular redox balance. Inhibition of sorbitol accumulation may be a potential method to improve the nuclear maturation of aged oocytes.

## INTRODUCTION

*In vitro* maturation (IVM) of immature oocytes has been applied to improve the clinical utility of oocytes, decrease the risk of ovarian hyperstimulation syndrome (OHSS) and increase the chances of fertility preservation [1]. However, the maturation rate of oocytes undergoing IVM is still significantly lower than that of *in vivo*-matured oocytes [2]. Coordination of nuclear maturation and cytoplasmic maturation in oocytes is required for successful fertilization and embryo development, but IVM usually fails to achieve synchronization of nuclear and cytoplasmic maturation [3]. The success of IVM systems also depends on the medium composition and culture conditions [4]. Therefore, many researchers have sought to modify the medium composition to improve the quality of *in vitro*-matured oocytes.

Social trends, including a greater concentration on education and careers, have driven women to delay childbirth [5]. However, reproductive capacity declines as women approach their later reproductive years, and increasing age is always associated with reductions in fecundity and infertility in the contexts of both natural fertility and assisted reproductive technology (ART) [6]. The live birth rate (LBR) per cycle of *in vitro* fertilization (IVF) is directly related to the age of the mother. The likelihood of ongoing pregnancy in women over 30 years of age decreases by approximately 1.5% per year [7]. The main causes of reproductive capacity decline are a decreasing number of oocytes and an increasing proportion of poor-quality oocytes, which result from ovarian aging. The ovarian function and oocyte pool of women decrease with age, while the percentage of abnormal oocytes, such as aneuploidy, increases [8]. Ovarian aging is associated with the decreased levels of anti-Müllerian hormone (AMH) and the antral follicular count (AFC), but an increased level of the follicle-stimulating hormone (FSH).It is also characterized by diminished ovarian reserve (DOR) in the clinic [9]. In addition to ovarian aging, a physiological process that naturally occurs with increasing age, “postovulatory aging” can also occur. Matured oocytes arrested at the metaphase stage of the second meiosis (M II) wait for fertilization after ovulation. However, if fertilization fails during the optimal fertilization period, oocytes will undergo a time-dependent decrease in quality whether they remain *in vivo* or cultured *in vitro* [10]. Previous studies have indicated that oocyte aging is associated with poor oocyte quality, whether naturally occurring with increasing age or during postovulatory aging. For example, the risk of a variety of defects, including the abnormal structure of the meiotic spindle and chromosomal aneuploidies, increases [11]. The function and structure of mitochondria in oocytes change after oocyte aging, which is accompanied by a decrease in the efficiency of ATP production and redox imbalance in oocytes [12]. In addition, oocyte apoptosis increases because of molecular changes, such as hyperactivation of caspase-3 [13]. Although several underlying causes have been proposed to explain the decreased quality of aged oocytes, perturbation of oocyte metabolism is likely one of the key factors [14]. To improve the quality of aged oocytes *in vivo* and *in vitro*, an increasing number of studies have focused on oocyte metabolism and its regulation [15].

Oocyte maturation requires an enormous amount of energy to synchronize nuclear and cytoplasmic maturation. Glucose is the major energy source during oocyte development and maturation, but energy is also provided by the cellular metabolism of lipids and proteins [16]. Four glucose metabolic pathways are found in the cumulus-oocyte complex (COC): glycolysis, the pentose phosphate pathway (PPP), the hexosamine biosynthesis pathway (HBP) and the polyol pathway [17]. The main glucose metabolic pathways of COCs are glycolysis and the PPP, which provide metabolites such as pyruvate and nicotinamide adenine dinucleotide phosphate reduced form (NADPH) that can be transferred to oocytes for utilization through gap junctions [18]. The most important mechanisms of ATP production in oocytes are the mitochondrial tricarboxylic acid cycle (TCA) and electron transfer; pyruvate for these processes comes from the cumulus cells surrounding oocytes. The polyol pathway can convert glucose to sorbitol via aldose reductase (AR) and can oxidize sorbitol to fructose through sorbitol dehydrogenase (SDH) [19]. However, since AR has a lower affinity for glucose than other enzymes, such as hexokinase, the polyol pathway accounts for a small percentage of glucose metabolism under normal glycemic conditions. Nevertheless, the polyol pathway is activated in a hyperglycemic environment as a result of saturation of the enzyme hexokinase [20]. Previous studies have indicated that NADPH consumption, sorbitol accumulation, and fructose and NADH generation play important roles in the etiology and complications of diabetes [21]. Recent studies have also shown that supplementation with sorbitol in IVM medium can restrain porcine oocyte maturation and subsequent embryo development by decreasing glutathione reduced form (GSH) levels and increasing reactive oxygen species (ROS) levels which indicated that sorbitol accumulation in cells is closely related to oxidative stress [22]. However, the precise roles of the polyol pathway in oocyte maturation and embryo development, especially in aged or aging oocytes, are not clear.

This study was designed to explore whether the polyol pathway is activated in aged oocytes and to elucidate the effects of increased sorbitol on the IVM procedure and quality of *in vitro*-matured oocytes. To understand the potential mechanism, the levels of oxidative stress and AR were tested. Our results suggest that inhibition of sorbitol production can improve the maturation rate of oocytes in aged mice during IVM and that the oxidative stress induced by sorbitol is a potential mechanism by which the polyol pathway affects oocyte maturation.

## RESULTS

### Sorbitol levels in the oocytes of aged mice

**Figure 1:**
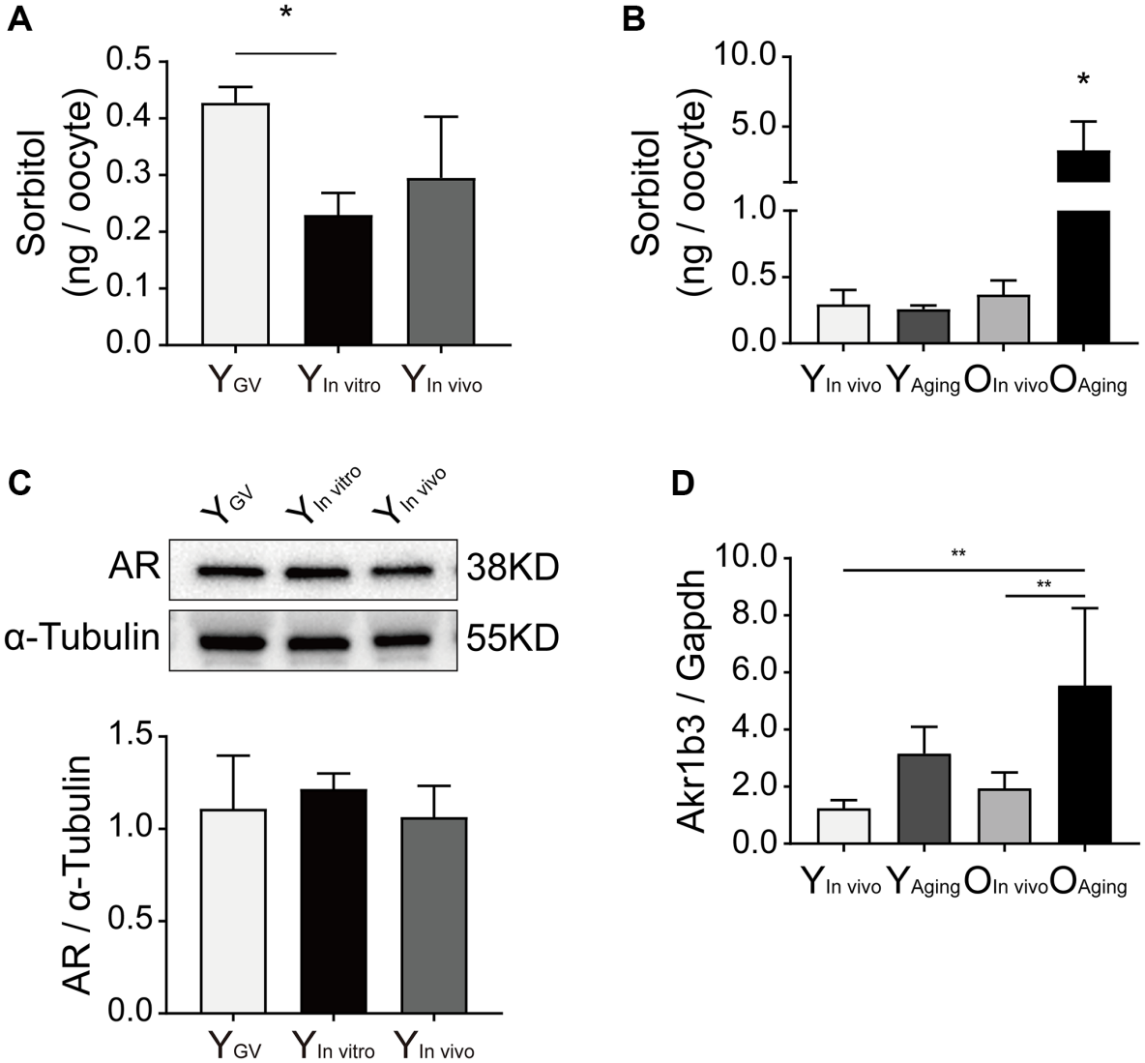
**Effect of sorbitol accumulation on oocytes of aged mice.** (**A**) The level of sorbitol in the IVM oocytes of young mice was significantly lower than that in the GV oocytes (*n* = 50). (**B**) The level of sorbitol in aged oocytes was significantly increased after *in vitro* aging (*n* = 50). (**C**) Effect of *in vitro* maturation on the expression of AR in the oocytes of young mice. Y_GV_ was the GV oocyte of young mice, Y_*In vitro*_ was the young IVM oocyte, and Y_*In vivo*_ was the young oocyte during *in vivo* natural maturation. (**D**) Effect of *in vitro* aging on the expression of Akr1b3 in the oocytes of aged mice. Y_Aging_ was the oocyte of young mice undergoing *in vitro* aging, O_*In vivo*_ was the aged oocyte during *in vivo* natural maturation, and O_Aging_ was the aged oocyte undergoing *in vitro* aging. All experiments were repeated at least three times. ^*^P < 0.05, ^**^P < 0.01.

The levels of sorbitol in oocytes were measured by HPLC/MS/MS. The sorbitol levels were significantly lower in the *in vitro*-matured oocytes of young mice than in germinal vesicle (GV)-stage oocytes (Y _GV_: 0.43 ± 0.03 vs. Y _*In vitro*_: 0.23 ± 0.04 vs. Y _*In vivo*_ 0.30 ± 0.11 ng/oocyte, *n* = 3, *P* < 0.05; Figure 1A). Interestingly, the sorbitol levels in the oocytes of aged mice were significantly increased after *in vitro* aging (O _aging_: 3.31 ± 2.05 vs. O _*in vivo*_: 0.37 ± 0.11 vs. Y _aging_: 0.26 ± 0.03 vs. Y _*in vivo*_: 0.30 ± 0.11 ng/oocyte, *n* = 3, *P* < 0.05; Figure 1B). AR is the key enzyme associated with intracellular sorbitol production in the polyol pathway, and the Akr1b3 gene encodes AR. There were no significant differences in the expression of AR in the oocytes of young mice (*P* > 0.05; Figure 1C). However, the relative expression of Akr1b3 was significantly increased in aged oocytes after *in vitro* aging (Akr1b3/Gapdh: O _aging_: 5.55 ± 2.71 vs. O _*in vivo*_: 1.95 ± 0.55 vs. Y _aging_: 3.16 ± 0.93 vs. Y _*in vivo*_: 1.24 ± 0.29, *P* < 0.01; Figure 1D).

### Maturation, fertilization and subsequent blastocyst formation of IVM oocytes of aged mice

**Figure 2:**
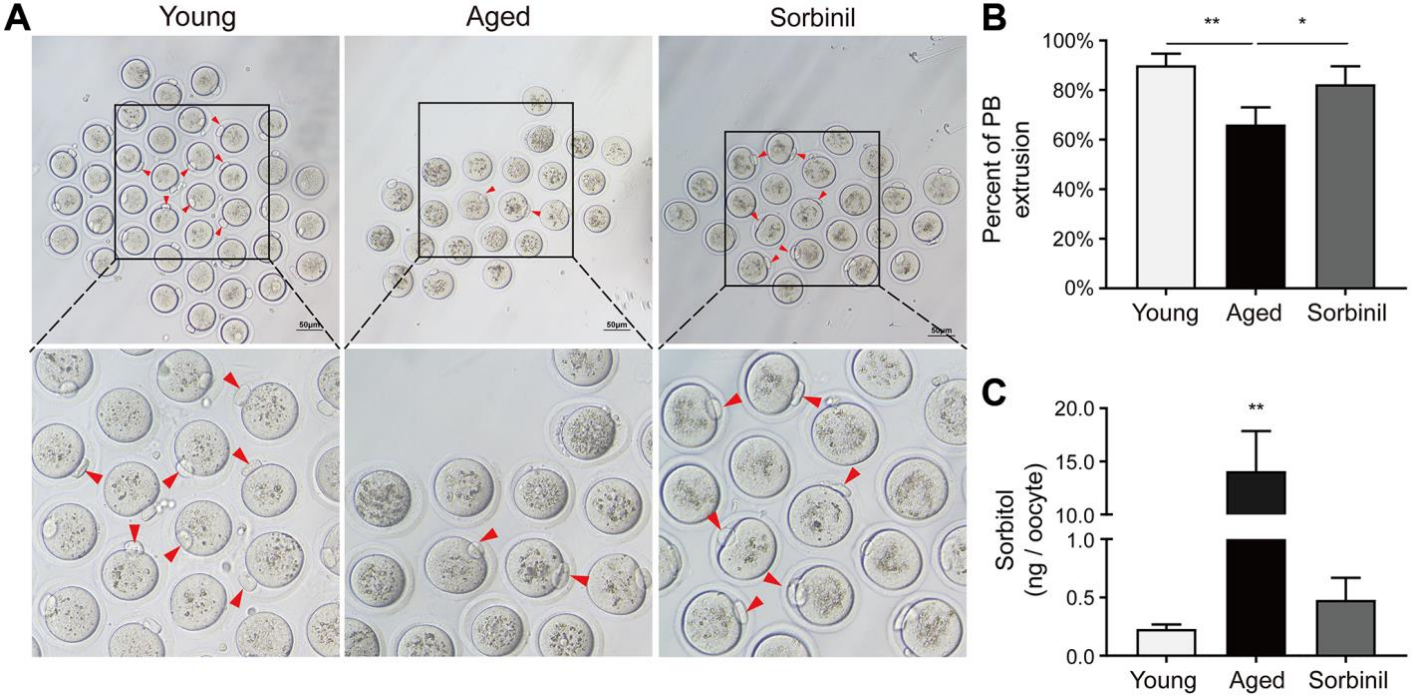
**The *in vitro* oocyte maturation of aged mice.** (**A**) The *in vitro* maturation of oocytes of aged mice and young mice and the effect of sorbinil as an inhibitor of AR. The red arrows in the images identify oocytes that extruded the first polar body (PB). (**B**) The first polar body extrusion in aged oocytes (aged) was increased by sorbinil. Each group had at least 20 oocytes. (**C**) The level of sorbitol in aged IVM oocytes was significantly higher than that in young IVM oocytes (*n* = 50). The level of sorbitol in aged IVM oocytes was decreased by sorbinil (sorbinil), suggesting the inhibitory effect of sorbinil on sorbitol accumulation. All experiments were repeated at least three times. Scale bar = 50 μm, ^*^P < 0.05, ^**^P < 0.01.

**Figure 3:**
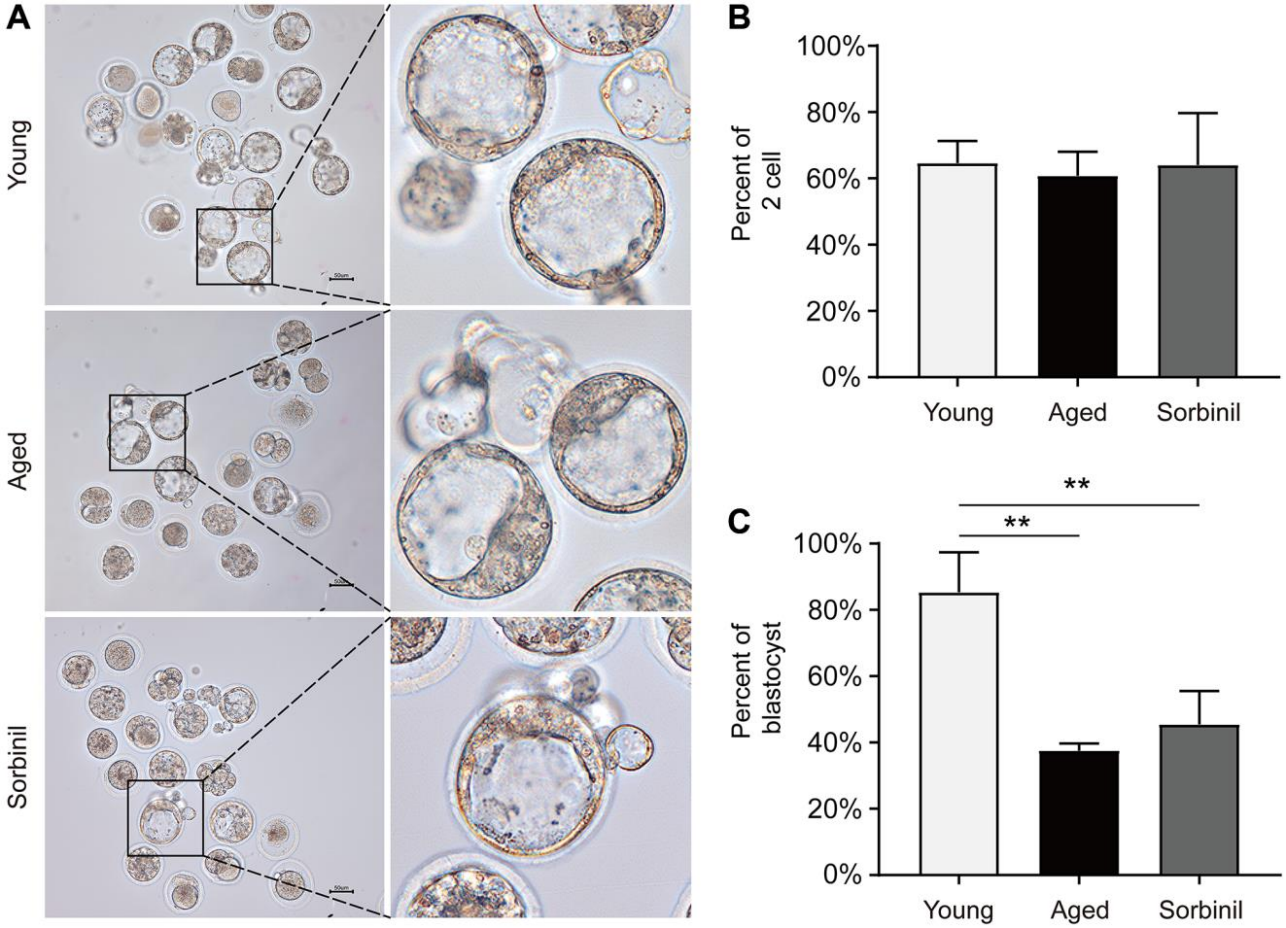
**Fertilization and subsequent blastocyst formation of IVM oocytes of aged mice.** (**A**) Early embryo development of IVM oocytes after fertilization. (**B**) The rates of 2-cell embryos (the number of 2-cell embryos/the total number of oocytes) in young IVM oocytes (young), aged IVM oocytes (aged), and aged IVM oocytes treated with sorbinil (sorbinil). (**C**) The rates of blastocyst formation (the number of blastocysts/the number of 2-cell embryos) in the above three groups. All experiments were repeated at least three times. Scale bar = 50 μm, ^**^P < 0.01.

Oocyte maturation was evaluated by observing the first polar body extrusion (PBE); specifically, the percentage of oocytes with PBE was used to reflect oocyte nuclear maturation. The maturation rate of oocytes undergoing IVM of aged mice (9 months old) was significantly lower than that of young mice (8 weeks old) (66.1% ± 6.9% vs. 90.1% ± 4.6%, *n* = 3, *P* < 0.01; Figure 2A and 2B). The sorbitol levels in the oocytes of aged mice were significantly higher than those in the oocytes of young mice (14.08 ± 3.78 vs. 0.23 ± 0.04 ng/oocyte, *n* = 3, *P* < 0.01; Figure 2C). Sorbinil, a specific inhibitor of AR, was used to inhibit the production of intracellular sorbitol. The sorbitol levels in the IVM oocytes of aged mice were significantly decreased when 200 μM sorbinil was used in the IVM medium (14.08 ± 3.78 vs. 0.48 ± 0.19 ng/oocyte, *n* = 3, *P* < 0.01; Figure 2C), and the maturation rate of oocytes undergoing IVM was increased upon sorbinil supplementation (82.4% ± 7.2% vs. 66.1% ± 6.9%, *n* = 3, *P* < 0.05; Figure 2B). COCs were cultured for 16–18 h in IVM medium, and the expanded COCs were collected for IVF. Fertilization and blastocyst formation were then observed, and there were no significant differences in the percentages of 2-cell embryos (%) among the young, aged and sorbinil treatment groups (64.6% ± 6.7%, 60.8% ± 7.3%, and 64.0% ± 15.7%, *n* = 3, *P* > 0.05; Figure 3A and 3B). The rate of blastocyst formation in the young group was significantly higher than that in the aged groups (85.3% ± 12.0% vs. 37.6% ± 2.1%, *n* = 3, *P* < 0.01; Figure 3C). The rate of blastocyst formation was partially rescued by sorbinil in aged oocytes. However, there was no difference between the aged group and the sorbinil group (37.6% ± 2.1% vs. 45.4% ± 10.1%, *n* = 3, *P* > 0.05; Figure 3C).

### Oxidative stress related to excessive sorbitol in aged oocytes

**Figure 4:**
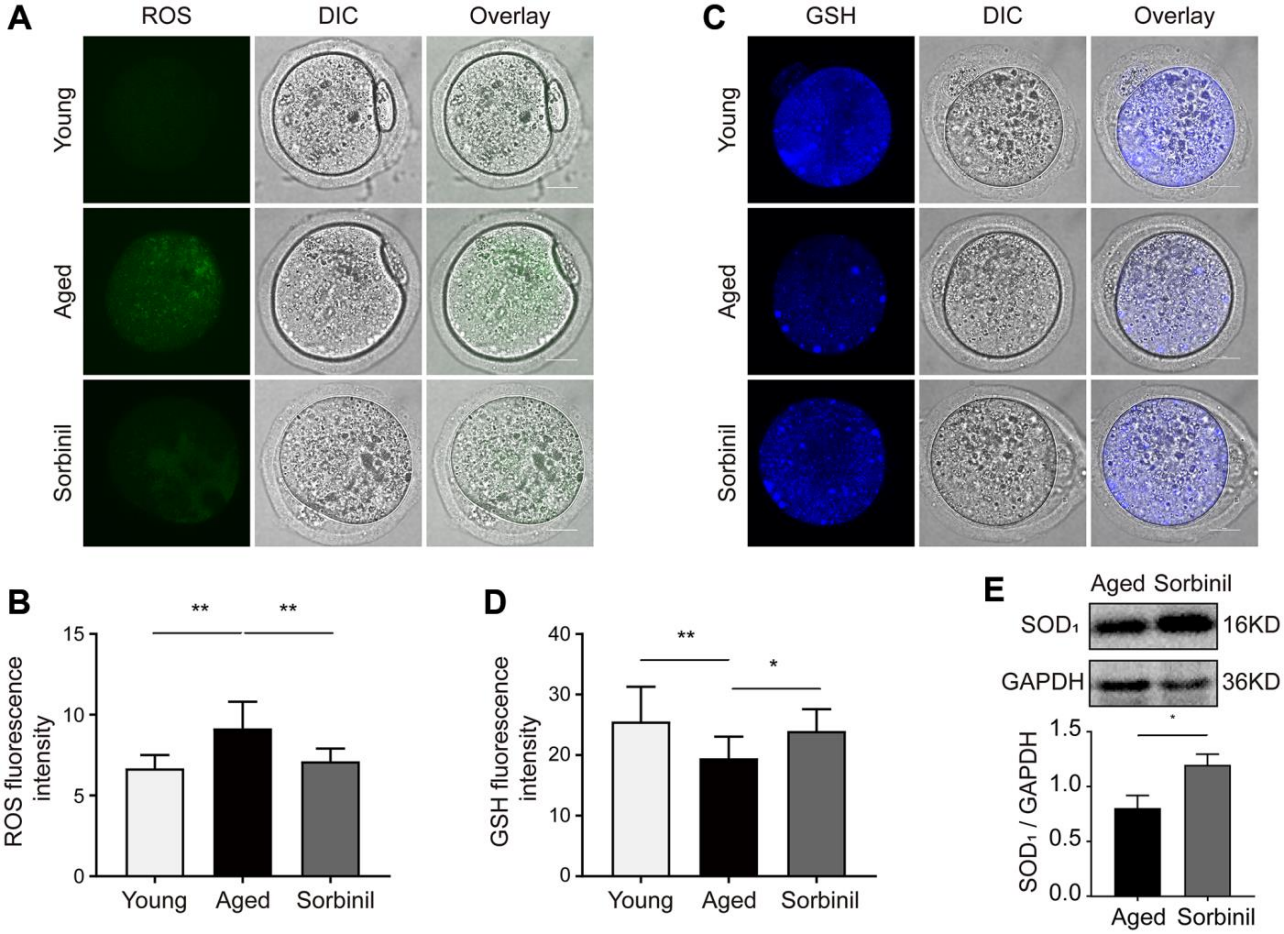
**Effects of sorbitol accumulation on the levels of ROS and GSH and the expression of SOD1 in IVM oocytes.** (**A**) The levels of ROS in young IVM oocytes (young), aged IVM oocytes (aged), and aged IVM oocytes treated with sorbinil (sorbinil). Fifteen oocytes in each group from three independent experiments were tested. (**B**) The differences in ROS levels in the above three groups. Fifteen oocytes in each group from three independent experiments were tested. (**C**) The levels of GSH in above three groups. (**D**) The differences in GSH levels in the above three groups. (**E**) The expression of SOD1 in the IVM oocytes. All experiments were repeated at least three times. Scale bar = 50 μm, ^*^P < 0.05, ^**^P < 0.01.

ROS levels were tested with carboxy-DFFDA, a type of fluorescent probe. The ROS level in the aged group was significantly higher than that in the young group (*n* = 15, *P* < 0.01; Figure 4A and 4B), while the ROS level in the sorbinil treatment group was lower than that in the aged group (*n* = 15, *P* < 0.01; Figure 4A and 4B). The GSH levels in aged oocytes were significantly lower than those in young oocytes (*n* = 15, *P* < 0.01; Figure 4C and 4D), while sorbinil treatment increased the GSH levels in aged oocytes (*n* = 15, *P* < 0.05; Figure 4C and 4D). The expression of SOD1 in oocytes was also tested by Western blot analysis. Interestingly, the expression of SOD1 was lower in the aged group than in the sorbinil group (SOD1/GAPDH: 0.81 ± 0.12 vs. 1.21 ± 0.10, *n* = 3, *P* < 0.05, Figure 4E). Our results indicated that the expression of SOD1 in the IVM oocytes was higher in the young group than in the aged group (SOD1/GAPDH: 2.01 ± 0.29 vs. 0.81 ± 0.12, *n* = 3, *P* < 0.01, Supplementary Figure 1). We also found that the expression of SOD1 in the IVM oocytes was higher in the young group than in the sorbinil group (SOD1/GAPDH: 2.01 ± 0.29 vs. 1.21 ± 0.10, *n* = 3, *P* < 0.05, Supplementary Figure 1).

### Hyperactivation of the polyol pathway is related to the poor quality of aged oocytes

**Figure 5:**
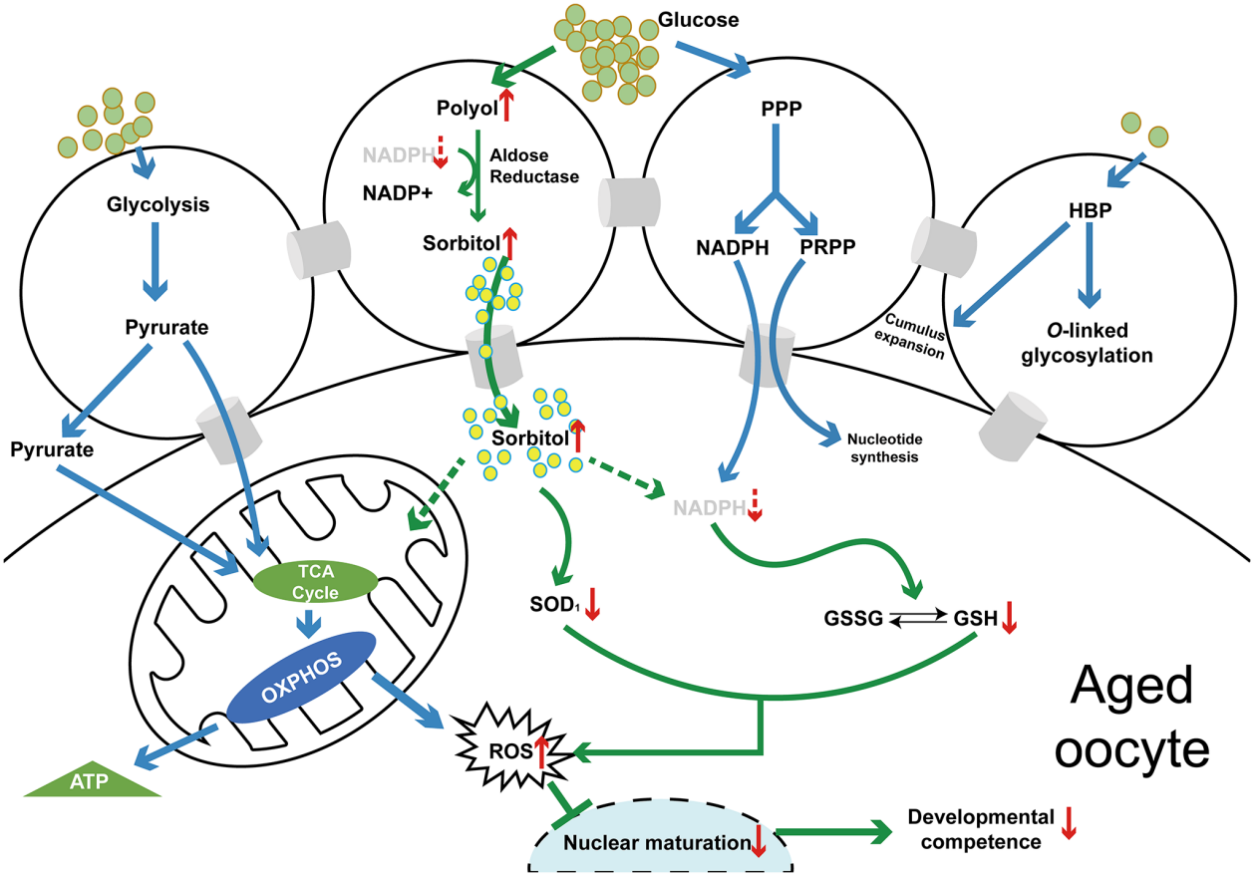
**A schematic diagram of the polyol pathway in COC and the mechanism of the effect of sorbitol accumulation on oocyte quality (*in vivo* or *in vitro*) of aged mice.** Abbreviations: PPP: pentose phosphate pathway; HBP: hexosamine biosynthesis pathway; TCA: tricarboxylic acid cycle; OXPHOS: oxidative phosphorylation; NADPH: nicotinamide adenine dinucleotide phosphate (reduced form); PRPP: 5-phosphoribosyl-1-pyrophosphate; GSSG: glutathione (oxidized form); GSH: glutathione (reduced form); ROS: reactive oxygen species.

Together, the results indicated that hyperactivation of the polyol pathway induces excessive production of sorbitol in aged oocytes, and sorbinil, as a specific inhibitor of AR, effectively reduces sorbitol accumulation in oocytes. Excessive sorbitol and oxidative stress may interact as both cause and effect (Figure 5). Increased levels of ROS and decreased levels of GSH and SOD1 were found in aged oocytes, suggesting that oxidative stress is related to sorbitol accumulation, which results in the poor quality of aged oocytes.

## DISCUSSION

In the present study, we first explored the effects of the polyol pathway on *in vitro*-matured oocytes from aged mice. Our results showed that sorbitol accumulation during IVM significantly inhibited oocyte nuclear maturation in aged mice and that sorbinil supplementation in IVM medium improved the nuclear maturation of oocytes by restraining sorbitol accumulation.

Sorbitol levels were increased in the IVM oocytes of aged mice, but we did not examine the sorbitol levels in the IVM medium. A previous study indicated that the polyol pathway is activated and that sorbitol accumulates in diabetic mice; the same study also indicated that sorbinil supplementation in IVM medium can increase the GV breakdown (GVBD) percentage in oocytes [23]. Similarly, Karen Uhde et al. [24] found that the sorbitol levels in maturation medium after bovine oocytes were cultured *in vitro* for 8 h were 5.9 times higher than those at 0 h. Increasing evidence has shown that AR expression is associated with sorbitol levels in mice [19]. Our results also indicated that the expression of AR was not significantly increased in young oocytes during IVM (*P* > 0.05) and that the mRNA expression of AR was significantly increased in aged oocytes during *in vitro* aging. This suggests that sorbitol accumulation in aged IVM oocytes is induced by the highly activated polyol pathway. The activity of AR and SDH during the formation of sorbitol accumulation may therefore be more important than their respective expression. However, since AR has a much lower affinity for glucose than other enzymes and the activities are easily interfered with by *in vitro* conditions, direct *in vitro* measurement of the activities of AR and SDH is not feasible. The expression level of AR is usually used to evaluate the polyol pathway and sorbitol accumulation in cells. Interestingly, the sorbitol levels in young IVM oocytes were still lower than those in aged oocytes. We did not test the expression of SDH due to a lack of monoclonal antibodies. Zhang et al. proposed that sorbitol production is eliminated by SDH via the polyol pathway in liver cells with high activation [25].

The quality of oocytes is important for fertilization and subsequent embryo development. *In vitro* culture conditions may be unable to fully support oocyte metabolism and adequate ATP production, which may partially explain the suboptimal success rates of current IVM systems compared with *in vivo* oocyte maturation [15]. A previous study showed that the glucose metabolism capacity in porcine COCs undergoing IVM is lower than that in porcine COCs matured *in vivo* [26]. However, the differences in glucose metabolism, especially in the polyol pathway, between IVM oocytes and *in vivo*-matured oocytes were not clarified, and the differences between young oocytes and aged oocytes undergoing the same IVM procedure were even less clear. In the present study, we did not observe changes in the fertilization rate or blastocyst formation rate after sorbinil supplementation in the IVM medium of aged oocytes. Various factors could decrease the rates of aged oocytes in addition to oxidative stress, such as the high incidence of aneuploidies and mitochondrial dysfunction. In addition, the synchronization of nuclear and cytoplasmic maturation is critical for fertilization and subsequent embryo development. Although our results indicated that supplementation with sorbinil in IVM media decreased sorbitol accumulation in aged oocytes and improved nuclear maturation during IVM, cytoplasmic maturation may play more important roles in blastocyst formation in aged oocytes, and sorbinil supplementation in IVM media simply reduced the sorbitol level and was not sufficient to completely rescue the poor quality of aged oocytes. In the present study, activation of the polyol pathway during IVM suppressed the maturation of aged oocytes, and sorbinil supplementation improved the maturation rate of aged oocytes by inhibiting sorbitol accumulation.

The sorbitol accumulation induced by the activated polyol pathway results in intracellular redox imbalance, increases the NADH/NAD^+^ ratio in the cytoplasm and decreases cytosolic NADPH levels [27]. The decreases in NADPH levels are due to competition of AR with the antioxidant enzyme GSH reductase for the same pool of cytoplasmic NADPH [28, 29]. NADPH downregulation can induce alterations that lead to intracellular redox imbalance, as GSH is required for the maintenance of intracellular redox balance in the pathogenesis of diabetic complications [30]. We found that sorbitol pathway activation and sorbitol accumulation in aged oocytes increased ROS levels and decreased GSH levels, consistent with a previous report showing that sorbitol supplementation in IVM medium is associated with decreased GSH and increased ROS levels in oocytes [22]. However, in a previous study, the effect of sorbitol on oocyte maturation was not clear in young porcines, and supplementation with high-dose sorbitol in IVM media decreased the percentage of MII oocytes. In our study, we focused on the effect of excessive endogenous sorbitol produced during IVM in the oocytes of aged mice. As a result of the high level of oxidative stress in aged oocytes, activation of the polyol pathway could aggravate the negative impact of oxidative stress on aged oocyte quality. Our results also indicated that sorbinil supplementation in IVM medium decreased ROS levels by inhibiting sorbitol accumulation in aged IVM oocytes. A previous study showed that, in BV-2 cells, sorbinil inhibits the production of ROS [31]. There is also increasing evidence that ROS levels in IVM oocytes are associated with the expression of antioxidant proteins [32, 33], and sorbinil has been indicated to activate antioxidant defense against ROS produced in response to high glucose exposure [34]. We found that, in IVM oocytes, especially aged IVM oocytes, the expression of SOD1 was increased by sorbinil supplementation in IVM medium via inhibition of sorbitol accumulation. In addition to GSH and SOD1, more factors are likely involved in the link between increased oxidative stress and sorbitol accumulation and decreased oocyte quality. Although how sorbitol impacts GSH levels and SOD1 expression was not investigated in detail, our results suggest that sorbitol accumulation inhibits oocyte maturation by disrupting the intracellular redox balance. In fact, the evaluation of oocyte quality is varied, including spindle morphology/chromosome alignment, organelles such as mitochondria, apoptosis or autophagy level, or DNA damage. In our previous study, postovulatory aging increased apoptosis and abnormal morphological changes of the spindle in oocytes. Additionally, the function of mitochondria decreased, and ROS accumulated in aging oocytes [35]. These results were consistent with those of other studies on oocyte postovulatory aging [36–38]. In addition, a previous study on the natural aging of oocytes also found that the oocytes of aged mice or women aged over 38 years had increasing percentages of abnormal spindles and mitochondria, which were similar to oocytes undergoing postovulatory aging [39]. However, in our present study, we did not repeat these experiments to evaluate the quality of aged oocytes. The hypothesis that sorbitol accumulation affects oocyte quality through oxidative stress needs further study in the future.

In summary, the polyol pathway in oocytes of aged mice can be further activated during IVM, which results in sorbitol accumulation. This phenomenon is not apparent in the oocytes of young mice, possibly because of the effect of SDH in the polyol pathway and the redox balance in the fresh oocytes of young mice. However, oxidative stress in aged oocytes is exacerbated due to sorbitol accumulation during IVM, which inhibits oocyte maturation. Sorbinil supplementation in the IVM medium of aged oocytes can improve oocyte maturation by inhibiting sorbitol accumulation, suggesting that inhibition of the polyol pathway could be a potential way to improve the maturation rate of aged oocytes during IVM.

## MATERIALS AND METHODS

### Antibodies and chemicals

The chemicals and other reagents used in this study were purchased from Sigma Chemical Co. (St. Louis, MO, USA) unless otherwise stated. Rabbit polyclonal anti-α-tubulin, anti-AR, and anti-SOD1 antibodies were purchased from Abcam Co. (Cambridge, UK).

### Animal care and welfare

The protocol of the animal experiment was approved by the Institutional Animal Care and Use Committee (IACUC) of Nanjing Medical University (protocol number IACUC-1903026). C57BL/6J mice (8 weeks and 9 months old) were purchased from Charles River Co. (Beijing, China) and kept in a facility free of specific pathogens.

### Oocyte *in vitro* maturation, *in vitro* aging and sorbinil treatment

C57BL/6J female mice (young mice, 8 weeks old; aged mice, 9 months old) were given 10 IU of pregnant mare serum gonadotropin by intraperitoneal injection. The mice were sacrificed by carbon dioxide euthanasia after 48 h, and the bilateral ovaries were transferred into HEPES-buffered M2. COCs were released by puncturing the antral follicles with a 1 ml injector. IVM medium was prepared according to the method in a previous report with a slight modification [40]. The IVM medium consisted of α-MEM (Gibco, New York, USA) supplemented with 5% FBS, 50 U/L r-hFSH, 3 ng/ml hEGF, 0.25 mM sodium pyruvate and 0.5% penicillin-streptomycin. The COCs were collected into a 20 μl microdrop of IVM medium (10 COCs per drop) and then incubated for 16–18 h at 37°C in humidified 5% CO_2_ in air. At 14 h after injection of 10 IU of HCG following PMSG injection, the COCs obtained from each oviduct were collected into a 20 μl microdrop of HEPES-buffered M2 medium (10 COCs per drop) and then cultured for 24 h at 37°C in humidified 5% CO_2_ in air. This procedure was considered *in vitro* oocyte aging (as the model of postovulatory aging). The oocytes were transferred into M2 medium supplemented with 80 IU/mL hyaluronidase solution (SAGE *In Vitro* Fertilization Inc., Trumbull, USA) for 1–2 min to isolate cumulus cells. Sorbinil, a specific inhibitor of AR, was used to inhibit the intracellular production of sorbitol. This compound has been applied for the treatment of diabetic complications in clinical practice [31]. Sorbinil was dissolved in DMSO to a concentration of 200 mM as a stock solution and stored at −20°C before use. The stock solution of sorbinil was then diluted to 200 μM and used to supplement IVM medium. The concentration of DMSO in the IVM medium did not exceed 0.1%. The dosage of sorbinil was selected on the basis of a previous study [23]. The groups were as follows: 9-month-old mice were considered the aged group, and 8-week-old mice were considered the control (young) group. The COCs from the aged group and the young group were cultured *in vitro* to obtain mature oocytes (IVM), which were described as IVM oocytes of aged mice and IVM oocytes of young mice, respectively. In the sorbinil treatment group, 200 μM sorbinil was added to the IVM medium of the aged oocytes. In this study, IVM oocytes were also compared with *in vivo*-matured oocytes in the aged group and the young group.

### Oocyte *in vitro* fertilization and blastocyst formation

IVF was conducted as previously described by Ling Li et al. [41]. Briefly, sperm were obtained by dissecting out the epididymis from C57BL/6J mice aged 10–20 weeks and cultured in TYH medium for 1 h for capacitation. The COCs were matured in IVM medium for 16–18 h and then transferred to HTF medium containing capacitated sperm. After fertilization in a 37°C incubator for 4–6 h, zygotes were cultured in a 20 μl microdrop of KSOM medium with 10 zygotes per drop at 37°C in an incubator containing 5% CO_2_, 5% O_2_, and 90% N_2_. Two-cell embryos were observed 23–43.5 h after fertilization, and blastocysts were observed 72–88.5 h after fertilization.

### Sorbitol level measurement by HPLC/MS/MS

HPLC/MS/MS was carried out as described by Klepacki et al. [42]. Before testing, optimization was performed using an Agilent 1290 UPLC system coupled to an AB Sciex Triple Quad 4500 system (Applied Biosystems Inc., Foster City, CA). Cogent diamond hydride columns (150 × 2.1 mm, 2.0 μm) were utilized for chromatographic separation of the sorbitol. The LC columns were maintained at 40°C in a thermostatically controlled column compartment. The solvents were 5 mM ammonium formate mixed with an aqueous solution containing 0.01% ammonia (mobile phase A) and acetonitrile (mobile phase B). The following gradient was employed: 0–1.5 min, 15% A; 1.5–5 min, 15% A-50% A; 5–7 min, 50% A; 7–8 min, 50% A-15% A; and 8–13 min, 15% A. The injection volume was 1 μl, and the flow rate was 0.2 ml/min. Electrospray ionization mass spectrometry was used with the following common parameters: temperature, 450°C; ion spray voltage, −4500 V; collision gas, medium; curtain gas, 10 psi; ion source gas 1, 45 psi; and ion source gas 2, 45 psi. The multiple reaction monitoring (MRM) detection mode was used. A 10 mg/ml stock solution of sorbitol was prepared by dissolving 0.04 g of sorbitol standard in 3.96 ml of 85% acetonitrile. Calibrators (16, 32, 64, 128, 250, 500 ng/ml) were obtained by diluting the stock solution. In total, 50 oocytes were collected per sample. The samples were added to 1 ml of methanol and extracted by ultrasound for 30 min. Then, they were centrifuged, and the supernatant was collected. The solid residue was extracted again with 1 ml of methanol and 1 ml of 50% methanol. The supernatants were combined, dried with nitrogen and reconstituted with 80% acetonitrile for testing.

### Intracellular ROS and GSH levels in oocytes

To test the levels of ROS and GSH in oocytes, 50-carboxy-20, 70-difluoro-dihydrofluorescein diacetate (carboxy-DFFDA; Thermo), a fluorescent probe capable of detecting powerful oxidants, such as H_2_O_2_ and peroxynitrite, and Cell Tracker Blue (4-chloromethyl-6.8-difluoro-7-hydroxycoumarin, CMF2HC; Invitrogen) were used. The oocytes were incubated in a 10 μM solution of carboxy-DFFDA or CMF2HC in M2 medium for 30 min at 37°C under 5% CO_2_ gas. The oocytes were then washed three times in M2 medium before they were transferred to a glass-bottom dish for confocal microscopy. The fluorescence intensities were analyzed using ImageJ software.

### Real-time PCR analysis

An RNeasy Plus Micro Kit (Qiagen, Germany) was used to extract the RNA. Ten oocytes were prepared for each sample. cDNA was produced by using a synthesis kit (Thermo, US), and Gapdh was used as the reference gene. The primer sequences were as follows: Akr1b3 forward: 5′-AACGTGATACCTAGTGACACCG-3′; reverse: 5′-AGCCAGGTTTGTTCAAGATCC-3′; Gapdh forward: 5′-GGTTGTCTCCTGCGACTTCA-3′; reverse: TGGTCCAGGGTTTCTTACTCC. A real-time PCR kit was used (Takara, US). The relative gene expression was calculated by the ΔΔCT method.

### Western blotting

Thirty oocytes per sample were prepared by adding 10 μl of 2× sample buffer, as described previously [35]. The samples were boiled for 5 min and loaded onto 10% Acr-Bis gels (Beyotime Biotechnology, Shanghai, China). The proteins were separated by SDS-PAGE and then transferred to PVDF membranes (Micron Separations, Westboro, MA). The membranes were blocked in TBS containing 0.1% Tween 20 and 5% low-fat dry milk for 2 h at room temperature and then incubated overnight at 4°C with rabbit anti-AR (1:500), anti-SOD1 (1:500) or anti-α-tubulin primary antibody (1:500). After three washes in TBS containing 0.1% Tween 20 and incubation with HRP-conjugated secondary antibodies at room temperature for 2 h, an enhanced chemiluminescence (ECL) kit was used to detect the signals. The bands were semiquantified with ImageJ software.

### Statistical analyses

Statistical analyses were performed using GraphPad Prism 7 software (GraphPad Software, US). All experiments were repeated at least three times, and each experimental group included at least 20 oocytes unless otherwise specified. The significance of the differences between groups was analyzed by one-way ANOVA, and Student’s *t* test was used to analyze the differences between two groups. The data are expressed as the mean ± SD, and differences with *P* values <0.05 were considered statistically significant.

### Data availability statement

The datasets used and/or analyzed during the current study are available from the corresponding author on reasonable request.

## Supplementary Materials

Supplementary Figure 1
