# Supplementary material for: Sorbitol accumulation decreases oocyte quality in aged mice by altering the intracellular redox balance
Source: Aging (Albany NY). 2021 Dec 12;13(23):25291–303. doi: 10.18632/aging.203747 (PMC8714154; doi:10.18632/aging.203747)
Supplement: Supplementary Figure 1 [file aging-13-203747-s001.pdf]

SUPPLEMENTARY FIGURE

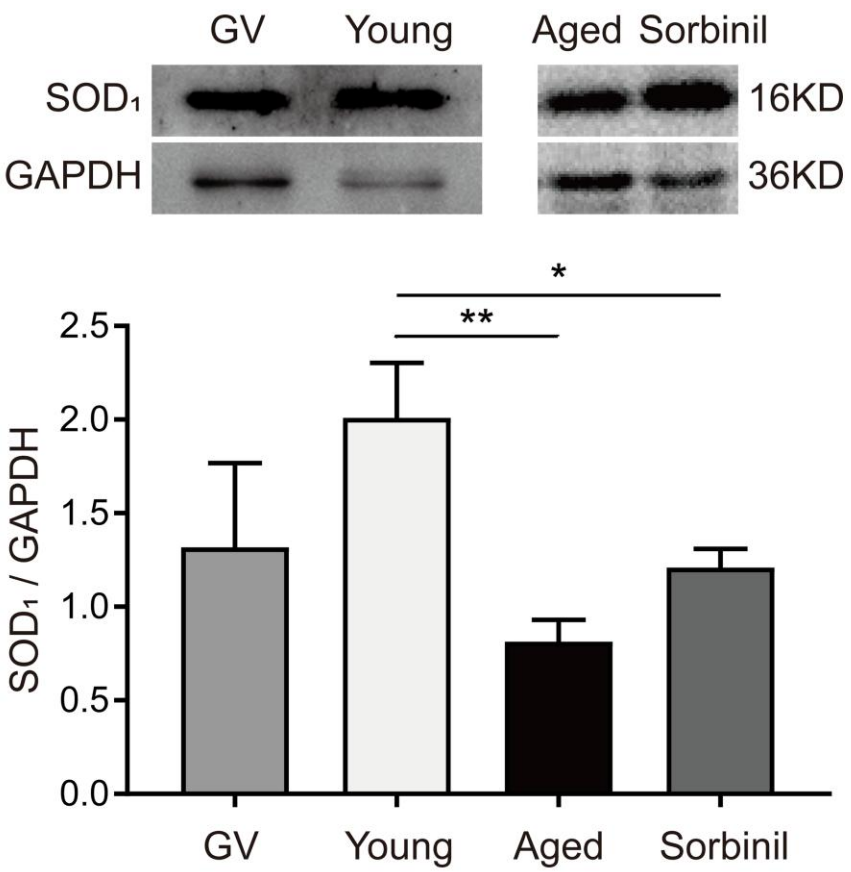

Supplementary Figure 1. SOD1 expression was downregulated in the aged IVM oocytes compared with the young IVM oocytes. The GV group was the GV oocytes from the young mice. The young group was the young IVM oocytes. The aged group was the aged IVM oocytes. The sorbinil group was the aged IVM oocytes with the sorbinil treatment. $^{*}P < 0.05$, $^{**}P < 0.01$.
